# The influence of carbapenem resistance on mortality in solid organ transplant recipients with *Acinetobacter baumannii* infection

**DOI:** 10.1186/1471-2334-12-351

**Published:** 2012-12-13

**Authors:** Erika Ferraz de Gouvêa, Ianick Souto Martins, Marcia Halpern, Adriana Lúcia Pires Ferreira, Samanta Teixeira Basto, Renato Torres Gonçalves, Beatriz Meurer Moreira, Guilherme Santoro-Lopes

**Affiliations:** 1 Infectious Disease Clinic, Hospital Universitário Clementino Fraga Filho, Universidade Federal do Rio de Janeiro, Rua Justiniano da Rocha 201/302, Vila Isabel, CEP 20551-010, Rio de Janeiro, RJ, Brazil; 2 Medicine School, Infectious Disease Section, Universidade Federal Fluminense, Rio de Janeiro, Brazil; 3 Clinical Microbiology Laboratory, Hospital Universitário Clementino Fraga Filho, Universidade Federal do Rio de Janeiro, Rio de Janeiro, Brazil; 4 Hepatology Clinic, Hospital Universitário Clementino Fraga Filho, Universidade Federal do Rio de Janeiro, Rio de Janeiro, Brazil; 5 Nephrology Clinic, Hospital Universitário Clementino Fraga Filho, Universidade Federal do Rio de Janeiro, Rio de Janeiro, Brazil; 6 Microbiology Institute, Centro de Ciências da Saúde, Universidade Federal do Rio de Janeiro, Rio de Janeiro, Brazil

**Keywords:** *Acinetobacter baumannii*, Resistance, Carbapenem, Transplantation, Mortality

## Abstract

**Background:**

Infection with carbapenem-resistant *Acinetobacter baumannii* has been associated with high morbidity and mortality in solid organ transplant recipients. The main objective of this study was to assess the influence of carbapenem resistance and other potential risk factors on the outcome of *A. baumannii* infection after kidney and liver transplantation.

**Methods:**

Retrospective study of a case series of *A. baumannii* infection among liver and renal transplant recipients. The primary outcome was death associated with *A. baumannii* infection. Multivariate logistic regression was used to assess the influence of carbapenem resistance and other covariates on the outcome.

**Results:**

Forty-nine cases of *A. baumannii* infection affecting 24 kidney and 25 liver transplant recipients were studied. Eighteen cases (37%) were caused by carbapenem-resistant isolates. There were 17 (35%) deaths associated with *A. baumannii* infection. In unadjusted analysis, liver transplantation (p = 0.003), acquisition in intensive care unit (p = 0.001), extra-urinary site of infection (p < 0.001), mechanical ventilation (p = 0.001), use of central venous catheter (p = 0.008) and presentation with septic shock (p = 0.02) were significantly related to a higher risk of mortality associated with *A. baumannii* infection. The number of deaths associated with *A. baumannii* infection was higher among patients infected with carbapenem-resistant isolates, but the difference was not significant (p = 0.28). In multivariate analysis, the risk of *A. baumannii*-associated mortality was higher in patients with infection acquired in the intensive care unit (odds ratio [OR] = 34.8, p = 0.01) and on mechanical ventilation (OR = 15.2, p = 0.04). Appropriate empiric antimicrobial therapy was associated with significantly lower mortality (OR = 0.04, p = 0.03), but carbapenem resistance had no impact on it (OR = 0.73, p = 0.70).

**Conclusion:**

These findings suggest that *A. baumannii*-associated mortality among liver and kidney transplant recipients is influenced by baseline clinical severity and by the early start of appropriate therapy, but not by carbapenem resistance.

## Background

Bacterial infections are among the most relevant causes of morbidity and mortality after organ transplantation. The high prevalence of multidrug-resistance among bacterial pathogens causing infections in solid organ transplant recipients is an additional concern [1,2]. In a recent report, infections with multidrug-resistant Gram-negative bacteria were associated with higher mortality among liver transplant recipients [3]. In this context, the emergence of carbapenem-resistant *Acinetobacter baumannii* (CRAB) as a significant pathogen in many transplant centers around the world is a special dilemma due to the limited therapeutic choices available to treat these infections [1,4]. Nonetheless, there are few data on the epidemiology of CRAB infection among solid organ transplant recipients and on its impact on the survival of these patients [5-9]. The objective of the present study was to analyze the possible influence of carbapenem resistance and other potential risk factors on mortality among solid organ transplant recipients with *A. baumannii* infection.

## Methods

### Study design

This was a retrospective study of a case-series of kidney transplant (KT) and liver transplant (LT) patients with infection by *A. baumannii*. A case-patient was defined as a subject with *A. baumannii* infection according to Centers of Disease Control and Prevention criteria [10]. The study was approved by the Institutional Committee on Research Ethics of the Hospital Universitário Clementino Fraga Filho/Medicine School of the Universidade Federal do Rio de Janeiro.

The primary endpoint was death associated with *A. baumannii* infection, and the secondary endpoint was death by any cause within 30 days of infection onset. Death was defined as associated with *A. baumannii* infection if the patient had persistent manifestations of the infection and no other possible cause of death was detected.

### Case finding and data collection

An electronic search was performed in the microbiology database to identify potential case-patients from January 2002 to January 2009. Data on the following variables were collected from patients’ medical records: sex; age; use of antimicrobial drugs within the previous 3 months; the use of hemodialysis, mechanical ventilation and of central venous catheters at the time of *A. baumannii* infection diagnosis; treatment for acute graft rejection with methyl-prednisolone pulse therapy or anti-thymocyte globulin within the previous three months; date of hospital and intensive care unit (ICU) admission; site and date of diagnosis of *A. baumannii* infection, and the use of appropriate empiric antibiotic therapy. This was defined as the start of an effective antimicrobial drug within 48 hours after the diagnosis of infection. Antimicrobial therapy with polymyxin B was presumed to be effective for cases of CRAB infection although susceptibility test for this antibiotic was not routinely performed. The date of infection was defined as that when the first clinical specimen with isolation of *A. baumannii* was collected. Infection was defined as acquired in hospital or in the ICU if occurred >48h after hospital or ICU admission, respectively. All patients were followed until death or hospital discharge.

Bacterial identification and antimicrobial susceptibility testing were performed by the VITEK-1 system (BioMérieux Vitek Inc, Hazelwood, USA). Susceptibility to imipenem, gentamicin, amikacin, ampicillin/sulbactam, piperacillin-tazobactam, cefotaxime, ceftazidime, cefepime, ciprofloxacin, minocycline and trimethoprim/sulfamethoxazole was determined by disk-diffusion according to CLSI recommendations [11]. As of July 2006, isolates were also tested for meropenem susceptibility. Isolates with intermediate susceptibility to imipenem or meropenem were also defined as carbapenem-resistant.

### Transplant protocols

Perioperative antimicrobial prophylaxis for kidney and liver transplantation consisted of cefazolin for 24 hours and ampicillin plus sulbactam for 48 hours, respectively. Immediately after transplantation, KT patients remained in the nephrology unit with a basic immunossupressive protocol consisting of a triple drug regimen including corticosteroids, a calcineurin inhibitor (usually tacrolimus) and mycophenolate mofetil or sirolimus. Dual drug regimens consisting of corticosteroids and mycophenolate mofetil or tacrolimus were prescribed for recipients of allografts from HLA-identical donors. Anti-thymocyte globulin was used as induction therapy in KT patients who underwent retransplantation.

After transplantation, LT patients were admitted to a surgical ICU where they remained until weaned from mechanical ventilation. Immunossupression for LT patients with chronic hepatitis B or C infection consisted of corticosteroids and a calcineurin inhibitor (tacrolimus or cyclosporin). Azathioprine or mycophenolate was additionally used for the immunossupression of patients with other indications for LT.

For both LT and KT patients, trimethoprim/sulfamethoxazole (80mg/400mg per day) was prescribed for six months to one year after transplantation to prevent *Pneumocystis jiroveci* infection.

### Statistical analysis

The Mann–Whitney test was used to compare the distribution of numeric variables while the Chi-square and Fisher’s exact tests were used to analyze categorical variables. Potential associations of the study variables with the outcomes were explored by univariate and multivariate analyses in logistic regression models. All covariates that were associated with a p value <0.25 in the univariate analysis were selected for the multivariate analysis. Final adjusted models were obtained with a forward stepwise regression approach. Due to its clinical relevance, the variable “carbapenem resistance” was selected for the multivariate analysis and retained in all models analyzed in the stepwise regression process. Other variables were added one by one. The selection of the variable to be included at each additional step was based on their p value calculated with the log-likelihood ratio test. Only variables associated with a p-value ≤0.10 were included in the model. Variables with a p-value >0.10 were removed from the adjusted models. Collinearity between variables was checked. The goodness of fit of the final adjusted models was assessed by the Hosmer-Lemeshow test. Additionally, the Akaike’s information criterion (AIC) was used to compare candidate multivariable models. The selected final adjusted model for each studied outcome was that with the lowest AIC. To analyze the discrimination of the adjusted models for the prediction of each outcome we calculated the area under the receiver-operating curve (ROC) that were plotted using the predicted values and observed events. All p values presented are bicaudate and a p < 0.05 was considered statistically significant. Statistical analyses were performed with SPSS for Windows 18.0 (*SPSS Inc*., Chicago, Illinois).

## Results

During the study, 800 subjects underwent kidney (562 patients) or liver transplantation (238 patients). *A. baumannii* infection was diagnosed in 49 patients (6.1%). The cumulative incidence was significantly higher among LT recipients (25 cases, 10.5%) than in KT recipients (24 cases, 4.3%, p < 0.001). The sites of infection included 17 blood stream infections (4 related to central venous catheter), 14 symptomatic urinary tract infections, 13 pneumonias (10 associated with mechanical ventilation), 5 surgical site infections (one superficial and 4 intra-abdominal). The median time from transplantation to the diagnosis of *A. baumannii* infection was not significantly different between LT (13 days, interquartile range [IQR]: 6–45 days) and KT (13.5 days, IQR: 7–137 days) recipients (p = 0.23). KT and LT subjects affected by *A. baumannii* infection differed significantly regarding several other aspects (Table 1). LT recipients presented a higher frequency of infections acquired in the ICU (p < 0.001), affecting a non-urinary site (p < 0.001) and associated with septic shock (p = 0.04), mechanical ventilation (p < 0.001) and central venous catheter use (p < 0.001). In addition, LT patients tended to be older (p=0.09) and to have a more frequent history of previous carbapenem therapy (p = 0.08). On the other hand, treatment for an acute rejection episode was more frequent among KT recipients (p < 0.001). These patients also had a significantly higher serum creatinine level (p = 0.009).

**Table 1:** **Distribution of the study variables in kidney transplant and liver transplant recipients with *Acinetobacter baumannii *infection**

**Variable**	**Liver transplant (total = 25)**	**Kidney transplant (total = 24)**	**p**
	**n (%)**	**n(%)**	
Male sex	16 (64)	13 (54)	0.48
Age^a^	56 (38–63)	44 (31–55)	0.09
Infection acquired in the ICU	22 (88)	2 (8)	<0.001
Previous use of any antibiotic^b^	11 (55)	11 (55)	1.0
Previous use of carbapenem ^b^	8 (40)	3 (15)	0.08
Non-urinary site of infection	24 (96)	11 (46)	<0.001
Primary bacteremia	9 (36)	8 (33)	
Pneumonia	11 (44)	2 (18)	
Surgical site infection	4 (16)	1 (4)	
Bacteremia ^c^	16 (64)	12 (50)	0.32
Central venous catheter	25 (100)	6 (25)	<0.001
Mechanical ventilation	21 (84)	4 (17)	<0.001
Serum creatinine (μmol/L ) ^a^	130 (90 – 200)	320 (120–500)	0.009
Hemodialysis	8 (32)	4 (17)	0.21
Treated for acute graft rejection	1 (4)	11 (46)	0.001
Septic shock	11 (44)	4 (17)	0.04
Appropriate empiric therapy for *A. baumannii* infection	17 (68)	12 (50)	0.20
Resistance to carbapenem	12 (48)	6 (33)	0.10

ICU – intensive care unit; ^a^ median (interquartile range); ^b^ Data available in 40 patients; ^c^ includes 11 cases of secondary bacteremia.

Eighteen infections (37%) were caused by CRAB. Susceptibility tests for meropenem were available in eight of these cases. Resistance to both imipenem and meropenem was observed in all of them. The median time after transplantation to the diagnosis of the infection was longer for CRAB (20 days, IQR: 9–299 days) than for carbapenem susceptible *A. baumannii* infections (9 days; IQR: 5–27 days). The proportion of infections caused by CRAB was higher among LT recipients, but the difference was not statistically significant (p = 0.10, Table 1). Table 2 presents the distribution of other variables between cases of CRAB and carbapenem susceptible *A. baumannii* infection. CRAB infections were significantly associated with previous use of any antibiotic (p = 0.001) and, specifically, carbapenem (p < 0.001). CRAB infections were also significantly more frequent in patients with use of central venous catheter (p = 0.005) and in subjects undergoing hemodialysis (p = 0.01).

**Table 2:** **Distribution of the study variables in patients with carbapenem resistant and carbapenem susceptible *Acinetobacter baumannii *infections**

**Variable**	**Carbapenem-Resistant (total = 18)**	**Carbapenem-Susceptible (total = 31)**	**p**
	**n = 18 (%)**	**n = 31 (%)**	
Male sex	8 (44)	21 (68)	0.11
Age^a^	48 (28–63)	48 (37–58)	0.94
Infection acquired in the ICU	11 (61)	13 (42)	0.20
Previous use of any antibiotic ^b^	14 (88)	8 (33)	0.001
Previous use of carbapenem ^b^	10 (63)	1 (4)	<0.001
Non-urinary site of infection	14 (78)	21 (68)	0.45
Primary bacteremia	5 (28)	12 (39)	
Pneumonia	6 (33)	7 (23)	
Surgical site infection	3 (17)	2 (6)	
Bacteremia ^c^	9 (50)	19 (61)	0.44
Central venous catheter	16 (89)	15 (48)	0.005
Mechanical ventilation	12 (67)	13 (42)	0.10
Serum creatinine (μmol/L) ^a^	200 (110 – 290)	230 (110–460)	0.39
Hemodialysis	8 (44)	4 (13)	0.01
Therapy for acute graft rejection	2 (11)	10 (32)	0.10
Septic shock	8 (44)	7 (23)	0.11
Appropriate empiric therapy for *A. baumannii* infection	10 (56)	19 (61)	0.69

ICU – intensive care unit; ^a^ median(interquartile range); ^b^ data available in 40 patients; ^c^ includes 11 cases of secondary bacteremia.

A total of 19 (39%) patients died: 18 within 30 days, and one on the 52^nd^ day after the diagnosis of the infection. This was the case of a liver transplant recipient who was reoperated on the first postoperative week because of a biliary fistula that was successfully repaired with a choledochojejunostomy. On the 28^th^ postoperative day, she was reoperated due to an abdominal abscess caused by *Stenotrophomonas maltophilia*. On the 50^th^ postoperative day she was reoperated again to drain another abdominal abscess. CRAB was isolated in the clinical specimen collected during this surgery. Despite prolonged treatment with polymyxin B combined with meropenem, she underwent laparotomy in two other occasions due to the persistence of the abdominal infection. CRAB was still isolated from the abscess secretion collected during the last reoperation, which took place one week before her death.

In 17 cases (35%), deaths were defined as associated with *A. baumannii* infection, including the one occurring 52 days after the infection. Most *A. baumannii*-associated deaths (15 cases, 88%) occurred within 14 days of the diagnosis. The median time from the diagnosis of infection to death tended to be shorter among patients who did not receive appropriate empiric antimicrobial therapy (one day) than in other patients (8 days, p = 0.07).

In unadjusted logistic regression analyses (Table 3), liver transplantation (p = 0.003), acquisition in ICU (p = 0.001), extra-urinary site of infection (p < 0.001), mechanical ventilation (p = 0.001), use of central venous catheter (p = 0.008) and presentation with septic shock (p = 0.02) were significantly related to a higher risk of mortality associated with *A. baumannii* infection. The number of deaths associated with *A. baumannii* infection was higher among patients infected with CRAB, but the difference was not significant (p = 0.28). Unexpectedly, therapy for acute graft rejection was associated with lower mortality (p = 0.03). This finding probably reflects confounding, as acute rejection was diagnosed more frequently among KT recipients who presented with lower baseline clinical severity as compared with LT recipients. Univariate regression analyses with overall 30-day mortality as the outcome yielded similar results (Table 4).

**Table 3:** **Univariate analyses of risk factors for death associated with *Acinetobacter baumannii *infection**

**Variable**	**Deaths (n = 17)**	**Survivors (n = 32)**	**Odds ratio (95%CI)**	**p**
	**n (%)**	**n (%)**		
Male sex	9 (53)	20 (63)	0.68 (0.21 – 2.22)	0.52
Age ( each 10 years)	10 (59)	12 (38)	1.08 (0.74 – 1.55)	0.70
Liver transplant	14 (82)	11 (34)	8.91 (2.10 – 37.8)	0.003
Infection acquired in the ICU	15 (88)	9 (28)	19.2 (3.63 – 101.3)	0.001
Previous use of any antibiotic ^a^	5 (36)	17 (65)	0.29 (0.08 – 1.15)	0.08
Previous use of carbapenem ^a^	4 (29)	7 (27)	1.09 (0.26 – 4.62)	0.91
Extra-urinary site of infection	17 (100)	18 (56)	Undefined	<0.001
Primary bacteremia	7 (41)	10 (31)		
Pneumonia	7 (41)	6 (19)		
Surgical site infection	3 (18)	2 (6)		
Bacteremia ^b^	11 (65)	17 (53)	1.43 (0.42 – 4.81)	0.57
Central venous catheter	16 (94)	15 (47)	18.1 (2.14 – 153.6)	0.008
Mechanical ventilation	15 (88)	10 (31)	16.5 (3.16 – 86.3)	0.001
Serum creatinine (for each 100 μmol/L) ^c^	170 (80– 230)	270 (110 – 460)	0.68 (0.44 – 1.06)	0.10
Hemodialysis	6 (35)	6 (19)	2.36 (0.62 – 8.97)	0.21
Therapy for acute graft rejection	1 (6)	11 (34)	0.17 (0.03 – 0.88)	0.03
Septic shock	9 (53)	6 (19)	4.88 (1,33 – 17.9)	0.02
Appropriate empiric therapy	8 (47)	21 (66)	0.47 (0.14 – 1.55)	0.21
Resistance to carbapenem	8 (47)	10 (30)	1.96 (0.58 – 6.56)	0.28

ICU – intensive care unit; CI = confidence interval; ^a^ Data available for 40 cases; ^b^ includes 11 cases of secondary bacteremia; ^c^ median(interquartile range).

**Table 4:** Univariate analyses of risk factors for overall 30-day mortality

**Variable**	**Deaths (n = 18)**	**Survivors (n = 31)**	**Odds ratio (95%CI)**	**p**
	**n (%)**	**n (%)**		
Male sex	10 (56)	19 (61)	0.79 (0.24 – 2.56)	0.69
Age (each 10 years)	10 (56)	12 (39)	0.97 (0.68 – 1.38)	0.85
Liver transplant	15 (83)	10 (32)	10.5 (2.46 – 44.8)	0.001
Infection acquired in the ICU	16 (89)	8 (26)	12.2 (2.83 – 52.7)	0.001
Previous use of any antibiotic ^a^	6 (40)	16 (64)	0.38 (0.10 – 1.40)	0.14
Previous use of carbapenem ^a^	5 (33)	6 (24)	1.58 (0.39 – 6.50)	0.52
Extra-urinary site of infection	17 (94)	18 (58)	12.4 (1.45 – 100.0)	0.02
Primary bacteremia	7 (39)	10 (32)		
Pneumonia	8 (44)	5 (16)		
Surgical site infection	2 (11)	3 (10)		
Bacteremia ^b^	12 (67)	16 (52)	1.65 (0.49 – 5.52)	0.42
Central venous catheter	17 (94)	14 (45)	20.6 (2.44 – 175.0)	0.005
Mechanical ventilation	15 (83)	10 (32)	10.5 (2.46 – 44.8)	0.001
Serum creatinine (for each 100 μmol/L) ^c^	200 (100 – 250)	220 (110 – 460)	0.76 (0.52 – 1.11) ^d^	0.16
Hemodialysis	7 (39)	5 (17)	3.31 (0.86 – 12.7)	0.08
Therapy for acute rejection	2 (11)	10 (32)	0.28 (0.07 – 1.16)	0.08
Septic shock	10 (56)	5 (16)	4.17 (1.15 – 15.0)	0.03
Appropriate empiric therapy	11 (50)	18 (58)	0.79 (0.24 -2.56)	0.69
Resistance to carbapenem	9 (50)	9 (29)	2.44 (0.73 – 8.17)	0.15

ICU – intensive care unit; CI = confidence interval; ^a^ Data available for 40 cases; ^b^ includes 11 cases of secondary bacteremia; ^c^ median(interquartile range).

In multivariate analyses (Table 5), ICU-acquired infection (p = 0.01) and mechanical ventilation at baseline (p = 0.04) were significantly related to a higher risk of death associated with *A. baumannii* infection. Oppositely, appropriate empiric antimicrobial therapy was associated with a significantly lower *A. baumannii*-associated mortality (p = 0.03). Acquisition in the ICU was the only factor significantly related to overall 30-day mortality (p = 0.001). In both multivariate analyses, resistance to carbapenem had no impact on mortality. The exclusion of resistance to carbapenem from the multivariate analysis did not cause appreciable changes in the results of the final adjusted model for each outcome (data not shown). The discrimination of the final multivariate model to predict death associated with *Acinetobacter baumannii* infection, as estimated by the area under the ROC curve, was 0.901 (95% confidence interval [CI]: 0.781 to 0.968). The area under the ROC curve calculated for the adjusted model for overall 30-day mortality was 0.799 (95% CI: 0.667 to 0.931).

**Table 5:** **Variables associated with mortality among liver and kidney transplant recipients with *A. baumannii *infection in multivariate logistic regression analysis**

**Models**	**Odds ratio (95% CI)**	**p**
***A. baumannii-associated mortality***^***a***^		
Infection acquired in the ICU	34.8 (2.05 - 593.1)	0.01
Mechanical ventilation	15.2 (1.22 – 189.2)	0.04
Appropriate empiric therapy	0.04 (0.002 – 0.74)	0.03
Resistance to carbapenem	0.73 (0.12 – 4.47)	0.70
***Overall 30- day mortality***^***b***^		
Infection acquired in the ICU	11.5 (2.61 – 49.8)	0.001
Resistance to carbapenem	1,93 (0.48 – 7.85)	0.36

CI: confidence interval; ICU: intensive care unit.

^a^ p = 0.89 in the Hosmer-Lemeshow test; ^b^ p = 0.94 in the Hosmer-Lemeshow test.

## Discussion

The emergence of infections caused by CRAB and other multi-drug resistant bacteria among transplant patients poses a difficult therapeutic challenge. In CRAB infections, therapeutic options are frequently restricted to drugs such as polymyxins and amikacin, which are associated with a higher risk of toxicity. Tigecycline has a better toxicity profile, however, its use as a monotherapy for the treatment of severe infections caused by CRAB, such as bacteremia and hospital-acquired pneumonia, is considered clinically unreliable [1,12]. Such therapeutic limitations justify the hypothesis that CRAB infection might be associated with a poorer prognosis especially in immunocompromised hosts.

Most studies that addressed the impact of *A. baumannii* infection on mortality defined overall 14 or 30-day mortality as their primary outcome. However, it has been observed both in transplant recipients [8] and in other critically ill patients [13] that CRAB infection may have a prolonged course that may ultimately result in death after several weeks of continued antimicrobial therapy [8]. Thus, the analysis of overall 14-day mortality might underestimate the impact of CRAB on mortality. On the other hand, overall 30-day mortality is more likely to be influenced by factors other than CRAB infection, like those related to the underlying clinical severity of the affected patients. Therefore, death associated with *A. baumannii* was defined as the primary outcome of this study. As expected, most *A. baumannii*-associated deaths occurred within 14 days. However, some patients had more prolonged course of the disease, and in one of these cases *A. baumannii*-associated death occurred 52 days after the diagnosis of infection.

There was no significant association between carbapenem resistance and mortality. It might be argued that this study was underpowered to detect a moderate increase in CRAB associated mortality. In fact, there was a non-significant increase in mortality among patients with CRAB infection in the univariate analyses. However, in the final multivariable model for death associated with *A. baumannii* infection, the estimated odds ratio for carbapenem resistance was lower than one. This finding suggests that the lack of association between carbapenem resistance and mortality in this report is unlikely to be related to inadequate statistical power. Conflicting results have been reported regarding the possible influence of carbapenem resistance on mortality among critically ill patients [14-19]. The reasons for this inconsistency are not clear, but it is probable that it comes from methodological differences among these studies, especially in the approach to minimize confounding by baseline clinical severity and to assess the effect of early appropriate therapy [19].

Acquisition of infection in the ICU and being on mechanical ventilation at the time of infection diagnosis were both strongly related to *A. baumannii*-associated death. These factors are probably surrogate markers of clinical severity and its association with mortality is in line with the results of other studies that pointed out the influence of factors related to baseline clinical severity on the outcome of *A. baumannii* infections [13,14,17,20,21].

The beneficial impact of early start of effective therapy on the outcome *A. baumannii* infection has been suggested by the results of other studies [6,18-22]. Indeed, Kwon at al. observed that carbapenem resistance was related to mortality in patients with *A. baumannii* bacteremia only if the use of effective therapy was not taken into account in the analysis [19]. Accordingly, in this series of abdominal organ transplant recipients, the use of appropriate empiric antimicrobial therapy was related to a significantly better outcome. Similar results have been reported by Kim and cols. [6] in a small case series of living-donor liver transplant recipients. However, that study also included subjects infected with *Acinetobacter lwoffii*, a species that has been associated with low mortality in immunocompromised patients [23]. It is plausible that the impact of delaying appropriate antimicrobial therapy for these infections may be higher in immunocompromised hosts than in immunocompetent patients. However, this issue was not addressed in the present study. Similarly to what has been described elsewhere [6,18,19,21,22], the proportion of patients who received inappropriate empirical antibiotic therapy was high in this series. Our findings underscore the importance of finely adjusting the selection of empiric antimicrobial therapy to the local epidemiological conditions.

The epidemiology and outcome of *A. baumannii* infection among KT recipients is largely unknown. We found that the overall cumulative incidence of *A. baumannii* infection was significantly lower in KT recipients than in LT patients. The prevalence of carbapenem resistance was not significantly different between isolates from both groups of patients, but clinical severity at the time of infection diagnosis was lower among KT patients as indicated by significantly lower frequency of ICU-acquired infection, use of central venous catheter, mechanical ventilation and extra-urinary site infection. As expected, the diagnosis of acute graft rejection was more frequent among KT recipients, but this factor was not independently related to *A. baumannii*-associated mortality in our analyses. The lower baseline clinical severity was probably responsible for the better outcome of KT patients that was observed only in the unadjusted statistical analysis. Unfortunately, because of the relatively small number of cases included in the study, we could not address specific prognostic factors for KT and LT recipients infected with CRAB.

The present study has some limitations. The sample size was relatively small and, thus, it is possible that the influence of some of the studied variables on mortality was missed in the multivariable analyses. Due to a retrospective design, we were not able to categorize patients according to any validated severity of illness score. Although other surrogate markers were used, we cannot rule out the possibility of residual confounding related to differences in baseline clinical severity. Similarly, it is not possible to preclude confounding by aspects related to antimicrobial therapy that were not addressed in the present study, such as the specific drug or combination of drugs used, doses and the exact number of hours elapsed between the clinical diagnosis of infection and the start of antibiotic treatment. One additional limitation of the study was the identification of *Acinetobacter* isolates by Vitek-1 system. However, most of the isolates would be classified as *A. baumannii* by the current, more accurate methods, and most human infections are caused by this species. Therefore, it is likely that this limitation did not have a major impact in the final results. Finally, because this is a single center study, the external validity of findings is uncertain. Larger, multicenter, prospective studies would help to provide a better definition of the factors that influence the outcome of *A. baumannii* infections in transplant recipients.

## Conclusions

These results suggest that *A. baumannii*-associated mortality among liver and kidney transplant recipients is influenced by baseline clinical severity and by the early start of appropriate therapy, but not by carbapenem resistance.

## Abbreviations

CRAB: Carbapenem-resistant *Acinetobacter baumannii*; KT: Kidney transplant; LT: Liver transplant; AIC: Akaike’s information criterion; ROC: Receiver operating curve; ICU: Intensive care unit; IQR: Interquartile range.

## Competing interests

The authors have no conflicts of interest to declare.

## Authors’ contributions

EFG, BMM and GSL designed the study and drafted the manuscript; ALPF carried out the microbiologic analysis; STB, MH and RTG were responsible for clinical data collection and helped to draft the manuscript; ISM in association with GSL performed data analysis. All authors read and approved the final manuscript.

## Pre-publication history

The pre-publication history for this paper can be accessed here:

http://www.biomedcentral.com/1471-2334/12/351/prepub
